# Prevalence and sequence of chronic conditions in older people with dementia: a multi-province, population-based cohort study

**DOI:** 10.24095/hpcdp.45.5.01

**Published:** 2025-05

**Authors:** Susan E. Bronskill, Azmina Artani, Laura C. Maclagan, Xuesong Wang, Hannah Chung, J. Michael Paterson, Andrea Gruneir, Karen A. Phillips, Rasaq Ojasanya, Xibiao Ye, Kayla McLean, Fernanda Ewerling, Claire Godard-Sebillotte, Victoria Massamba, Louis Rochette, Isabelle Vedel, Larry Shaver, Catherine Pelletier, Colleen J. Maxwell

**Affiliations:** 1 ICES, Toronto, Ontario, Canada; 2 Institute of Health Policy, Management and Evaluation, Dalla Lana School of Public Health, University of Toronto, Toronto, Ontario, Canada; 3 Sunnybrook Research Institute, Sunnybrook Health Sciences Centre, Toronto, Ontario, Canada; 4 Department of Family Medicine, Faculty of Medicine and Dentistry, College of Health Sciences, University of Alberta, Edmonton, Alberta, Canada; 5 Department of Health and Wellness, Chief Public Health Office, Government of Prince Edward Island, Charlottetown, Prince Edward Island, Canada; 6 Office of the Provincial Health Officer, Ministry of Health, Victoria, British Columbia, Canada; 7 School of Health Information Science, University of Victoria, British Columbia, Canada; 8 Department of Medicine and Health Sciences, Division of Geriatrics, McGill University, Montral, Quebec, Canada; 9 Bureau d’information et d’tudes en sant des populations, Institut national de sant publique du Qubec, Qubec, Quebec, Canada; 10 Department of Family Medicine, McGill University, Montral, Quebec, Canada; 11 Centre for Surveillance and Applied Research, Health Promotion and Chronic Disease Prevention Branch, Public Health Agency of Canada, Ottawa, Ontario, Canada; 12 Schools of Pharmacy and Public Health Sciences, University of Waterloo, Waterloo, Ontario, Canada

**Keywords:** dementia, chronic disease, comorbidity, epidemiology, hypertension, mental illness, osteoarthritis, pan-Canadian

## Abstract

**Introduction:**

Comorbid chronic conditions contribute to increased health service use and poor outcomes for people with dementia, but there is little information about the prevalence of these conditions in this population.

**Methods:**

We used linked administrative data from British Columbia (BC), Ontario (ON), Quebec (QC) and Prince Edward Island (PE) to identify a cohort of 287 453 individuals aged 65 years and older with prevalent dementia in April 2015, and followed this population until March 2020. We determined the prevalence of comorbid chronic conditions and ascertainment dates using Canadian Chronic Disease Surveillance System definitions, and used descriptive statistics to compare patterns across provinces.

**Results:**

Sociodemographic characteristics were similar across provinces (mean age: 83.0 [PE]–84.3 [BC] years; female sex: 61.8% [BC]–66.2% [QC]; and long-term care facility residence: 39.5% [QC]–41.6% [BC]). People with dementia commonly experienced five or more comorbid conditions (38.8% [PE]–53.5% [ON]); the most prevalent were hypertension (76.4% [PE]–81.4% [ON]), mental illness and alcohol- or drug-induced disorders (44.4% [QC]–91.2% [BC]) and osteoarthritis (43.8% [PE]–60.4% [ON]). Hypertension, diabetes and stroke were frequently apparent before dementia ascertainment, whereas heart failure and traumatic brain injury were apparent almost as frequently after dementia ascertainment as before.

**Conclusion:**

Patterns of comorbid chronic conditions were similar across provinces, with most present prior to dementia ascertainment. Health service planning strategies should be developed and shared across provinces to address the complex health care needs of people with dementia.

**Highlights**

- One-third to one-half of people with dementia also have five or more comorbid chronic conditions (from 38.8% in Prince Edward Island to 53.5% in Ontario).
- Common comorbid chronic conditions include hypertension (from 76.4% in Prince Edward Island to 81.4% in Ontario), mental illness and alcohol- or drug-induced disorders (from 44.4% in Quebec to 91.2% in British Columbia) and osteoarthritis (from 43.8% in Prince Edward Island to 60.4% in Ontario).
- Most chronic conditions, including hypertension, diabetes and stroke, were present before dementia was ascertained.
- A small number of chronic conditions, such as heart failure and traumatic brain injury, were present with equal frequency before and after dementia was ascertained.

## Introduction

Alzheimer disease and related dementias (referred to as dementia in this article) are progressive neurodegenerative diseases that affected 6.4% of Canadians aged 65years and older in fiscal year 2020 to 2021.^1^ The prevalence of dementia in Canada is rising, partly due to longer lifespans, and the number of individuals with dementia is expected to double by 2050, to 1.3million in Canada and 153 million globally.^2^ The large burden of cognitive and behavioural symptoms frequently associated with dementia is also stressful for people with the disease and their care partners.^3^ The direct health care costs for people living with dementia in Canada are projected to increase to CAD 18.2 billion by 2031.^4^

Because the number of chronic conditions increases with age and because specific conditions (such as diabetes and hypertension) are modifiable risk factors for dementia, people with dementia often have comorbid conditions.^5^ Comorbidity can exacerbate functional decline^5^ and accounts for significant additional health service use, fragmented care and poorer outcomes.^6^ Management of comorbid chronic conditions requires an integrated, interdisciplinary approach to improve the quality of life of people with dementia.^7^

Public health in Canada focusses on promoting health, preventing disease and injuries, responding to public health threats and providing information to support decision-making. Canada released its first national dementia strategy in 2019.^8^ One of the objectives of the strategy is prevention, which is supported by surveillance and data that provide a more accurate picture of the impact of dementia in Canada and ensure that preventive efforts are appropriately targeted.^8^ Surveillance includes tracking modifiable risk and protective factors and bridging evidence gaps.^8^

Describing the prevalence of comorbid chronic conditions, and their sequence of occurrence in relation to dementia case ascertainment, would help decision makers understand the complexity of the care required in order to plan services, to ensure the availability of adequate health care personnel and to educate health care providers on the management of comorbidity.^9^,^10^ However, there is limited information on the prevalence of chronic conditions among people with dementia across Canada. Our objective is to describe the prevalence of comorbid chronic conditions among older people with dementia, in four Canadian provinces, and to determine the temporal sequence of conditions relative to dementia ascertainment.

## Methods

***Study design and data sources***

We conducted a series of population-based, retrospective cohort studies of adults aged 65 years and older living in the provinces of British Columbia (BC), Ontario (ON), Quebec (QC) and Prince Edward Island (PE). These four provinces, where more than 75% of the Canadian population reside,^11^ participated in an initiative led by the Public Health Agency of Canada to report on the prevalence of comorbid chronic conditions among people with dementia. We used the health administrative databases available in each province to identify and characterize each cohort and to examine chronic conditions. These databases included provincial health insurance registries, the Canadian Institute for Health Information’s Discharge Abstract Database, physician billing claims and prescription drug dispensation databases (see Table 1). Database availability varied across provinces and some analyses used unique jurisdiction-specific datasets, such as the Ontario Mental Health Reporting System. 

**Table 1:** Summary of health administrative databases available by province, Canada

Database	Characteristics used in this study	Availability by province			
		BC	ON	QC	PE
Provincial health insurance registry	Age Sex Postal code (to report community size, neighbourhood income quintile and other area-based measures, where available via linkage to census data using Statistics Canada’s Postal Code Conversion File)	Yes	Yes	Yes	Yes
Canadian Institute for Health Information (CIHI) Discharge Abstract Database (DAD)	Acute care hospitalization within the 1 year prior to the index date Chronic conditions	Yes	Yes	Yes	Yes
Ontario Mental Health Reporting System (OMHRS)	Inpatient health service use for mental illnesses	NA	Yes	NA	NA
Physician billing claims	Outpatient visits to family physicians, dementia specialists and other specialists within the 1 year prior to the index date Chronic conditions	Yes	Yes	Yes	Yes
Prescription drug dispensations	Chronic conditions Note: Generally available for people aged ≥ 65 years and other select populations, e.g. long-term care residents, people receiving social assistance, people who do not have private drug coverage (QC)	Yes	Yes	Yes	Yes
Continuing Care Reporting System – Long-term care	Long-term care residents at the index date	Yes	Yes	NA^a^	NA
Canadian Index of Multiple Deprivation (CIMD)	Situational vulnerability and economic dependency quintiles	Yes	NA	NA	NA
Ontario Marginalization Index (ON-MARG)	Situational vulnerability and economic dependency quintiles	NA	Yes	NA	NA
Institut national de sant publique du Qubec (INSPQ) Material and Social Deprivation Index	Situational vulnerability and economic dependency quintiles	NA	NA	Yes	Yes

**Abbreviations: **BC, British Columbia; NA, not applicable; ON, Ontario; PE, Prince Edward Island; QC, Quebec. 

^a^ Information was derived from other health administrative databases using a validated algorithm. 

***Study population***

We identified all adults aged 65 years and older with dementia on 1 April 2015 (the index date) using the Canadian Chronic Disease Surveillance System (CCDSS) dementia case ascertainment algorithm,^12^ an adaptation of an algorithm previously validated in ON.^13^ The algorithm defines dementia as one or more hospital separation records (generated each time a patient is discharged or transferred from a health care facility, signs out against medical advice or dies); three or more physician claims for dementia within 2 years (at least 30 days between each); or one or more cholinesterase inhibitor drug prescriptions. The algorithm has a sensitivity of 79.3% and a specificity of 99.1% among older adults when validated against family physician records.^13^ An individual’s dementia ascertainment date was defined as the date they first met one of the criteria the algorithm uses.

We chose a study time period that excluded the COVID-19 pandemic in the follow-up period because of the changes that were occurring in health system use and health care service availability.

***Chronic conditions***

We identified 15 comorbid chronic conditions according to CCDSS algorithms based on hospital discharge abstracts, physician billing claims and dispensed medication claims (see Table 2 for the list of conditions and definitions).^12^ We selected conditions identified by the Lancet Commission report on dementia prevention, intervention and care^14^ as modifiable risk factors for dementia and by a 2017 study that used CCDSS data to examine multimorbidity,^15^ and based on the conditions’ associations with age.^16^ We also included traumatic brain injury based on a definition obtained from the literature,^17^-^19^ but as emergency department visit data were not available for all the participating provinces, case ascertainment was based solely on hospital records.

**Table 2:** Case ascertainment algorithms for chronic conditions based on CCDSS definitions

Chronic condition	Case definition	Case ascertainment date	ICD-9 codes	ICD-10 codes	Physician billing codes
Dementia	≥ 1 hospital separation records; or ≥ 3 physician claims within 2 years, with at least 30 days between qualifying claims; or ≥ 1 cholinesterase inhibitor drug prescription	Hospital separation record, last physician claim, or drug prescription date (whichever comes first)	046.1, 290.0, 290.1, 290.2, 290.3, 290.4, 294.1, 294.2, 331.0, 331.1, 331.5 (or 331.82 in ICD-9-CM)	G30, F00, F01, F02, F03	290, 331
Heart failure	≥ 1 hospital separation records or ≥ 2 physician claims within 1 year	Hospital separation record or last physician claim (whichever comes first)	428	I50	428
Hypertension (excluding gestational hypertension)	≥ 1 hospital separation records or ≥ 2 physician claims within 2 years	Hospital separation record or last physician claim (whichever comes first)	401, 402, 403, 404, 405	I10, I11, I12, I13, I15	401, 402, 403, 404, 405
Ischemic heart disease	≥ 1 hospital separation records or procedure codes, or ≥ 2 physician claims within 1 year	Hospital separation record or last physician claim (whichever comes first)	410, 411, 412, 413, 414^a^	I20, I21, I22, I23, I24, I25^a^	410, 411, 412, 413, 414
Diabetes (excluding gestational diabetes)	≥ 1 hospital separation records or ≥ 2 physician claims within 2 years	Hospital separation record or last physician claim (whichever came first)	250	E10, E11, E12, E13, E14	250
Stroke	≥ 1 hospital separation records or ≥ 2 physician claims within 1 year	Hospital separation record or last physician claim (whichever comes first)	325, 362.3x, 430, 431, 432.9, 433.x1, 434 (or 434.x1), 435.x, 436, 437.6^b^	G08, G45.x (excluding G45.4), H34.0, H34.1, I60.x, I61.x, I62.9, I63.x, I64, I67.6	325, 430, 431, 432.9, 434, 435, 436, 437.6
Traumatic brain injury^c^	≥ 1 hospital separation records	Date of hospital discharge or emergency department registration date (whichever came first)	310.2, 800.1, 800.3, 801.1, 801.3, 802.6, 802.7, 803.1, 803.3, 804.1, 804.3, 850, 851, 852, 853, 854, 907.0, 907.1, 925	F07.2, S02.0, S02.1, S02.3, S02.7, S02.8, S02.9, S06.0-S06.9, S07.1, T90.2, T90.5	Not applicable
Parkinsonism	≥ 2 physician claims within 1 year, with at least 30 days between qualifying claims	Last physician claim	Not applicable	Not applicable	332
Epilepsy	≥ 1 hospital separation records, or ≥ 3 physician claims within 2 years, with at least 30 days between qualifying claims	Hospital separation record or physician claim (whichever comes first)	345.0, 345.1, 345.4, 345.5, 345.6, 345.7, 345.8, 345.9	G40	345
Mental illness and alcohol- or drug-induced disorders (10-year look-back period)	≥ 1 hospital separation records or ≥ 1 physician claims within 1 year	Hospital separation record or physician claim (whichever comes first)	290.8, 290.9, 291–293, 294.0, 294.8, 294.9, 295–319	F04–F99	291–319 (50B in BC only)
Schizophrenia	≥ 1 hospital separation records, or ≥ 2 physician claims within 2 years, with at least 30 days between qualifying claims	Hospital separation record or physician claim (whichever comes first)	295	F20, F21, F23, F25	295
Osteoarthritis	≥ 1 hospital separation records, or ≥ 2 physician claims (separated by at least 1 day) within 5 years	Hospital admission or physician claim (whichever comes first)	715	M15–M19	715
Rheumatoid arthritis	≥ 1 hospital separation records, or ≥ 2 physician claims (> 8 weeks apart) within 2 years, with exclusion criterion^d^	730 days after hospital separation record or last physician claim (whichever comes first)	714	M05–M06	714
Osteoporosis	≥ 1 hospital separation records or ≥ 1 physician claims	Hospital separation record or last physician claim (whichever comes first)	733	M80, M81	733
Osteoporosis-related fractures (hip, forearm, pelvic, humerus and spine)	Hip: ≥ 1 hospital separation records (6-month episode) Forearm, pelvic and humerus: ≥ 1 hospital separation records or ≥ 2 physician claims within 3 months (6-month episode) Spine: ≥ 1 hospital separation records or ≥ 1 physician claims (6-month episode)^e^	Hip: Hospital admission Forearm, pelvic and humerus: Hospital admission or last physician claim (whichever comes first) Spine: Hospital admission or physician claim (whichever comes first)	Hip: 820 Forearm, pelvic and humerus: 813, 808, 805.6, 805.7, 812 Spine: 805.2–805.5	Hip: S72.0, S72.1, S72.2 Forearm, pelvic and humerus: S52, S32.1, S32.3, S32.4, S32.5, S42.2, S42.3, S42.4 Spine: S22.0, S22.1, S32.0	Hip: Not applicable Forearm, pelvic and humerus: 813, 814^f^, 808, 812 Spine: 805
Asthma	≥ 1 hospital separation records or ≥ 2 physician claims within 2 years	Hospital separation record or last physician claim (whichever comes first)	493	J45, J46	493
COPD	≥ 1 hospital separation records or ≥ 1 physician claims	Hospital separation record or physician claim (whichever comes first)	491, 492, 496	J41, J42, J43, J44	491, 492, 496

Abbreviations: CCDSS, Canadian Chronic Disease Surveillance System; COPD, chronic obstructive pulmonary disease; ICD-9, *International Classification of Diseases, 9th Revision*; ICD-9-CM, *International Classification of Diseases, 9th Revision, Clinical Modification*; ICD-10, *International Statistical Classification of Diseases and Related Health Problems, 10th Revision*; ICD-10-CA,*International Statistical Classiﬁcation of Diseases and Related Health Problems, 10th Revision, Canada.*

^a^ Percutaneous coronary intervention and coronary artery bypass graft coded in ICD-9-CM: 36.01, 36.02, 36.05, 36.10, 36.11, 36.12, 36.13, 36.14, 36.15, 36.16, 36.17, 36.19; the *Canadian Classification of Diagnostic, Therapeutic and Surgical Procedures*: 48.02, 48.03, 48.11, 48.12, 48.13, 48.14, 48.15, 48.16, 48.17, 48.19; and the *Canadian Classification of Health Interventions*: 1.IJ.50, 1.IJ.57.GQ, 1.IJ.54, 1.IJ.76. 

^b^ ICD-9 code 432.9 and ICD-10 code I62.9 were used to code hemorrhagic stroke prior to fiscal year 2015 to 2016, and ICD-10 code I61.9 has been used since then. 

^c^ Traumatic brain injury is not included in the CCDSS, but because of its relevance to dementia, we included it in this study. The case definition was obtained from the literature17-19 and does not include the emergency department visit criteria for case ascertainment as the data were not available for all jurisdictions. 

^d^ Subsequent to qualifying, cases with at least 2 physician claims (separated by at least 1 day) within 2 years with diagnoses of non-rheumatoid inflammatory arthritis including systemic autoimmune rheumatic diseases (ICD-9: 710; ICD-10-CA: M32.1, M32.8, M32.9, M33.x, M34.x, M35.1, M35.8, M35.9), polyarteritis nodosa and allied conditions (ICD-9: 446; ICD-10-CA: M30.x-M31.x), polymyalgia rheumatica (ICD-9: 725; ICD-10-CA: M35.3), psoriatic arthritis (ICD-9: 696; ICD-10-CA: L40.5, M07.0, M07.1 M07.2, M07.3), ankylosing spondylitis and other inflammatory spondylopathies (ICD-9: 720; ICD-10-CA: M45.x, M46.1, M46.8, M46.9) and arthropathy associated with other disorders classified elsewhere (ICD-9: 713; ICD-10-CA: M07.4, M07.5, M07.6) were excluded. People with psoriasis (ICD-9 code 696) were excluded because we cannot specifically exclude those with psoriatic arthritis given the number of digits required: the 2 physician claims must contain the same non-rheumatoid arthritis diagnostic codes at the 3-digit level for ICD-9(-CM) codes; the 2 physician claims must apply to all cases that qualified, i.e. those that qualified by way of a hospital separation or 2 physician claims; and excluded cases were excluded for the remainder of the study period. 

^e^ 6-month episode where any fracture codes during this period were considered part of the same event. The date of the first fracture code of a fracture event was used to establish the end point of the 6-month episode. 

^f^ One physician claim must include ICD-9 code 813 (or ICD-10-CA equivalent S52), but the other can include ICD-9 code 813 or 814 (or ICD-10-CA equivalent S52 or S62). 

Ascertainment for all conditions involved a look-back period, defined by data availability in each province and CCDSS case definitions,^12^ of approximately two decades except for mental illness and alcohol- or drug-induced disorders for which the look-back period was 10 years. Individuals were followed until 31 March 2020 for the development of chronic conditions and were censored on their death date, if applicable, to allow for a minimum follow-up of 5years after the study index date (1 April 2015). The unequal length of the look-back and follow-up periods reflect the natural progression of dementia, which tends to be ascertained later in life. The case ascertainment date for each condition was defined as the date individuals first met algorithm criteria.

***Other characteristics***

We used each province’s health insurance registry to obtain sociodemographic characteristics for individuals on the study index date, including their age, sex and postal code. The latter was linked to Statistics Canada’s Postal Code Conversion File to identify neighbourhood income quintiles and community size. The geocode, in turn, was linked to structural determinants of health indices that measure situational vulnerability and economic dependency. Neighbourhood income quintiles were defined based on the average household income of the neighbourhood (dissemination area) relative to the income level of the larger census regions.

We also identified health service utilization during the year preceding cohort index date. These included acute care hospitalizations, emergency department visits, family physician visits, dementia specialist visits (i.e. neurology, geriatrics and psychiatry) and other specialist visits.

***Statistical analyses***

We compared sociodemographic characteristics, prior health system use and prevalent comorbid chronic conditions across the provinces by reporting the mean (and standard deviation) for continuous variables (e.g. age) and counts and percentages for categorical variables (e.g. sex). To describe the temporal sequence of chronic conditions relative to dementia onset, we compared the number and percentage of chronic conditions ascertained before versus after dementia case ascertainment.

***Ethics approval***

In BC, this work was part of a comprehensive population health surveillance and research initiative of the Office of the Provincial Health Officer and was reviewed by the University of British Columbia Research Ethics Board (#H22-01818).

In ON, these datasets were linked using unique encoded identifiers and analyzed at ICES. ICES is an independent, nonprofit research institute whose legal status under Ontario’s health information privacy law allows it to collect and analyze health care and demographic data, without consent, for health system evaluation and improvement. The use of the data in this project is authorized under section 45 of Ontario’s *Personal Health Information Protection Act* and does not require review by a research ethics board.

In QC, this work is part of the ongoing chronic disease surveillance mandate assigned to the Institut national de sant publique du Qubec (INSPQ) by the Minister of Health and Social Services. All surveillance activities under this mandate are approved by the provincial Public Health Ethics Committee. No informed consent was required.

In PE, this work is part of ongoing and systematic chronic disease surveillance conducted by the Chief Public Health Office, under the authority of the Minister of Health and Wellness through sections 3 and 58 of the PE *Public Health Act*, and does not require review by a research ethics board.

## Results

**Figure 1:**
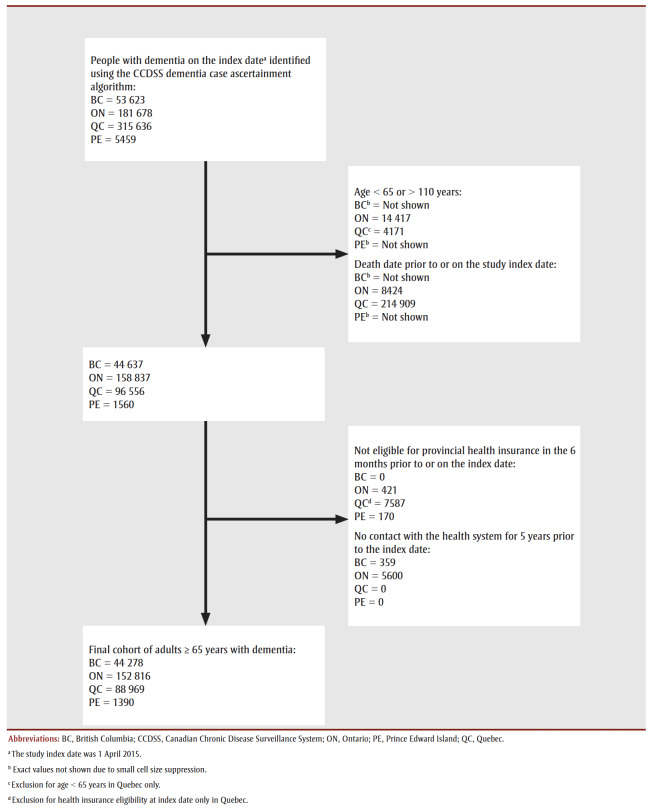
Flow diagram showing identification of people (≥ 65 years) with dementia in participating provinces, Canada

We identified 287 453 people aged 65 years and older with dementia living in the four participating provinces on 1 April 2015, from 1390 in PE to 152816 in ON. (For a flow diagram showing the process of identification, see Figure 1.)

***Baseline demographics***

The mean age of people with dementia was similar across the four provinces (from 83.0 years in PE to 84.3 years in BC) (Table 3). There were more females than males with the diagnosis in each province (from 61.8% in BC to 66.2% in QC). A large percentage of individuals were living in long-term care homes (from 39.5% in QC to 41.6% in BC) and in lower neighbourhood income quintiles 1 and 2 (from 47.7% in ON to 55.9% in PE). Fewer people with dementia were living in rural areas than in larger communities (from 10.5% in BC to 35.3% in PE). About one-fifth lived in areas with high situational vulnerability (from 15.1% in PE to 20.6% in ON).

**Table 3:** Baseline demographic and health service use characteristics of adults (≥ 65 years) with prevalent dementia on 1 April 2015, by province, Canada

Characteristics	BC N = 44 278		ON N = 152 816		QC N = 88 969		PE N = 1390	
	n	(SD) or %	n	(SD) or %	n	(SD) or %	n	(SD) or %
**Time since dementia case ascertainment, mean years (SD)**	3.6	(3.2)	4.3	(3.7)	3.8	(3.2)	2.8	(2.8)
**Mean age at index date,^a^ years (SD)**	84.3	(7.5)	83.3	(7.7)	83.4	(7.4)	83.0	(7.5)
Method of dementia case ascertainment, n (%)								
Hospitalization criterion	9087	20.5	6280	4.1	23 333	26.2	363	26.1
Physician claims criterion	17 543	39.6	44 964	29.4	18 452	20.7	381	27.4
Drug prescription criterion	17 648	39.9	101 572	66.5	47 184	53.0	646	46.3
Categorized age at index date, years								
65–69	1764	4.0	8309	5.4	3840	4.3	59	4.2
70–74	3736	8.4	13 949	9.1	7884	8.9	141	10.1
75–79	6558	14.8	23 176	15.2	13 542	15.2	241	17.3
80–84	10 300	23.3	35 208	23.0	21 761	24.5	324	23.3
85–89	11 416	25.8	38 312	25.1	23 311	26.2	355	25.5
≥ 90	10 504	23.7	33 862	22.2	18 631	20.9	270	19.4
Sex, n (%)								
Male	16 903	38.2	55 829	36.5	30 039	33.8	502	36.1
Female	27 375	61.8	96 987	63.5	58 930	66.2	888	63.9
Living in long-term care^a^	18 441	41.6	61 320	40.1	35 175	39.5	NA	NA
Neighbourhood income quintile								
Missing	284	0.6	816	0.5	NA	NA	6	0.4
Q1 (lowest)	12 846	29.0	39 295	25.7	NA	NA	360	25.9
Q2	8954	20.2	33 624	22.0	NA	NA	417	30.0
Q3	9199	20.8	28 705	18.8	NA	NA	272	19.6
Q4	6757	15.3	25 696	16.8	NA	NA	169	12.2
Q5 (highest)	6238	14.1	24 680	16.2	NA	NA	166	11.9
Community size, n								
Missing	281	0.6	779	0.5	252	0.3	6	0.4
≥ 1 500 000	21 358	48.2	59 490	38.9	41 942	47.1	0	NA
500 000–1 499 999	0	NA	25 753	16.9	17 927^b^	20.1	0	NA
100 000–499 999	11 431	25.8	35 222	23.0	17 927^b^	20.1	0	NA
10 000–99 999	6542	14.8	15 331	10.0	11 765	13.2	893	64.2
< 10 000 (rural)	4666	10.5	16 241	10.6	17 083	19.2	491	35.3
Situational vulnerability^c^								
Missing	88	0.2	1742	1.1	25 738	28.9	265	19.1
Q1 (least deprived)	9146	20.7	30 252	19.8	9399	10.6	153	11.0
Q2	9207	20.8	30 215	19.8	11 341	12.7	236	16.9
Q3	8448	19.1	29 154	19.1	11 869	13.3	188	13.5
Q4	9511	21.5	29 929	19.6	14 336	16.1	338	24.3
Q5 (most deprived)	7878	17.8	31 524	20.6	16 286	18.3	210	15.1
Economic dependency^c^								
Missing	88	0.2	1742	1.1	25 738	28.9	265	19.1
Q1 (least deprived)	5595	12.6	15 989	10.5	10 726	12.1	219	15.8
Q2	7008	15.8	20 005	13.1	10 582	11.9	117	8.4
Q3	7020	15.9	22 610	14.8	13 090	14.7	131	9.4
Q4	9322	21.1	27 395	17.9	14 052	15.8	343	24.7
Q5 (most deprived)	15 245	34.4	65 075	42.6	14 781	16.6	315	22.7
Previous health service use (at least once in the previous year)								
Acute care hospitalization	9294	21.0	37 322	24.4	23 140	26.0	415	29.9
Family physician visit	43 046	97.2	142 922	93.5	78 112	87.8	1153	82.9
Dementia specialist visit (including neurology, geriatrics and psychiatry)	10 580	23.9	44 296	29.0	23 307	26.2	465	33.5
Specialist visit, other	27 839	62.9	83 749	54.8	61 158	68.7	656	47.2

Abbreviations: BC, British Columbia; INSPQ, Institut national de sant publique du Qubec; ON, Ontario; Q, quintile; QC, Quebec; PE, Prince Edward Island; SD, standard deviation; NA, not available. 

^a^ Data availability varies across the participating provinces. For BC and ON, data were available from the Continuing Care Reporting System – Long-term care; for QC, information was derived from other health administrative databases using a validated algorithm; for PE, the data are not available. 

^b ^Community size is not available for less populous areas. 

^c^ Situational vulnerability quintiles and economic dependency quintiles for BC are from the Canadian Index of Multiple Deprivation (CIMD), for ON are from the Ontario Marginalization Index (ON-MARG) and for QC and PE are from the INSPQ Material and Social Deprivation Index. 

***Case ascertainment, health system use and comorbidity***

Time since dementia case ascertainment was similar across the four provinces, from 2.8 years in PE to 4.3 years in ON (Table 3). Use of cholinesterase inhibitor drug prescriptions was the most common data source for dementia case ascertainment across the provinces, although the percentage of cases identified by this data source varied from 39.9% in BC to 66.5% in ON.

The proportion of individuals with at least one acute care hospitalization in the year prior to dementia ascertainment was similar across the provinces, from 21.0% in BC to 29.9% in PE (Table 3). Family physician visits in the year prior to the index date were least prevalent in PE (82.9%) and most prevalent in BC (97.2%). In contrast, visits to dementia specialists were least prevalent in BC (23.9%) and most prevalent in PE (33.5%).

**Figure 2:**
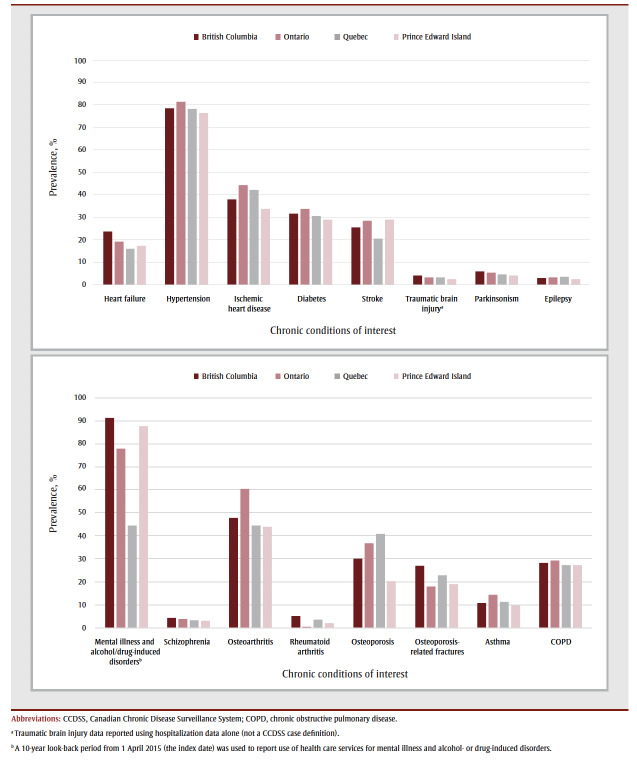
Prevalent chronic conditions among adults (≥ 65 years) with prevalent dementia on 1 April 2015, by province, Canada

Hypertension, mental illness and alcohol- or drug-induced disorders, and osteoarthritis were the most prevalent comorbid conditions (Figure 2; Table 4). The prevalence of mental illness and alcohol- or drug-induced disorders varied the most across the provinces, from 44.4% in QC to 91.2% in BC. The prevalence of ischemic heart disease, stroke, osteoporosis-related fractures, osteoarthritis, rheumatoid arthritis and osteoporosis also varied, but the relative ranking of prevalence was similar. Between 38.8% (PE) and 53.3% (ON) people with dementia had five or more comorbid chronic conditions.

**Table 4:** Prevalent chronic conditions among older adults (≥ 65 years) with prevalent dementia on 1 April 2015, by province, Canada

Characteristics, n (%)	BC N = 44 278		ON N = 152 816		QC N = 88 969		PE N = 1390	
	n	%	n	%	n	%	n	%
Number of chronic conditions								
≥ 5	21 900	49.5	81 492	53.3	42 440	47.7	539	38.8
Specific chronic conditions								
Heart failure	10 440	23.6	29 323	19.2	14 282	16.1	241	17.3
Hypertension	34 757	78.5	124 376	81.4	69 589	78.2	1062	76.4
Ischemic heart disease	16 801	37.9	67 660	44.3	37 620	42.3	468	33.7
Diabetes	13 965	31.5	51 560	33.7	27 114	30.5	402	28.9
Stroke	11 286	25.5	43 599	28.5	18 209	20.5	402	28.9
Traumatic brain injury	1766	4	5094	3.3	3016	3.4	33	2.4
Parkinsonism	2609	5.9	8428	5.5	4140	4.7	59	4.2
Epilepsy	1305	2.9	5018	3.3	3151	3.5	35	2.5
Mental illness and alcohol- or drug-induced disorders	40 382	91.2	119 083	77.9	39 532	44.4	1218	87.6
Schizophrenia	1842	4.2	5795	3.8	2824	3.2	42	3
Osteoarthritis	21 170	47.8	92 368	60.4	39 613	44.5	609	43.8
Rheumatoid arthritis	2258	5.1	753	0.5	3055	3.4	28	2
Osteoporosis	13 293	30	55 967	36.6	36 275	40.8	281	20.2
Osteoporosis-related fractures (hip, forearm, pelvic, humerus and spine)	11 957	27	27 342	17.9	20 369	22.9	262	18.9
Asthma	4728	10.7	21 842	14.3	9876	11.1	135	9.7
COPD	12 447	28.1	44 815	29.3	24 321	27.3	380	27.3

**Figure 3:**
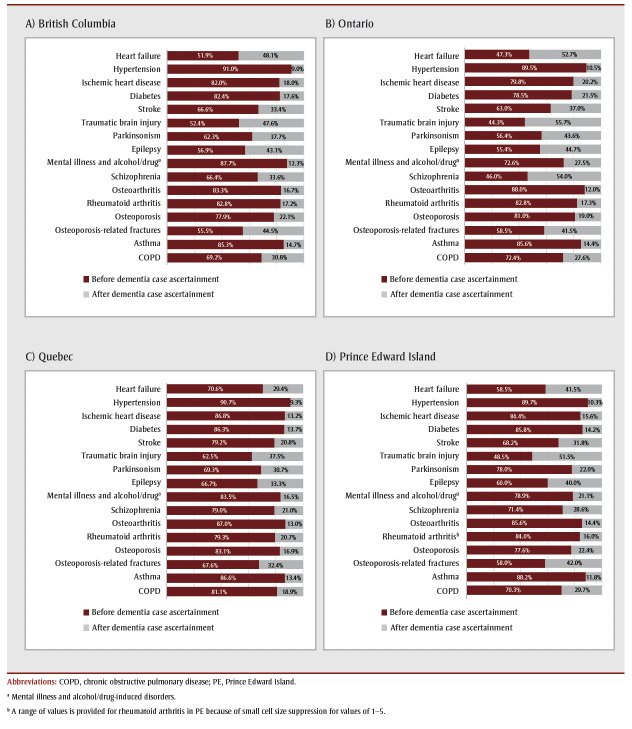
Sequence of chronic conditions apparent before and after dementia case ascertainment of adults (≥ 65 years) with prevalent dementia on 1 April 2015, by province, Canada

A larger percentage of chronic conditions were apparent prior to rather than after dementia case ascertainment (Figure 3). Vascular-related conditions such as hypertension, ischemic heart disease, diabetes and stroke as well as mental illness and alcohol- or drug-induced disorders, osteoarthritis, rheumatoid arthritis, osteoporosis and asthma were often apparent prior to dementia ascertainment. Heart failure, traumatic brain injury, parkinsonism, osteoporosis-related fractures and epilepsy became apparent almost as often after dementia ascertainment as before. These patterns were generally consistent across provinces, with some variation in prevalence estimates.

## Discussion

We compared the prevalence of comorbid chronic conditions among older adults with dementia across four Canadian provinces and examined their sequence of occurrence relative to dementia case ascertainment.

The COVID-19 pandemic revealed many issues in collecting, sharing, presenting and interpreting health data nationally.^20^ Federal and provincial governments are collaborating to develop a pan-Canadian strategy on health data management^21^ to which cross-provincial research contributes by consolidating the evidence base and refining methods to inform surveillance initiatives. Our methods may be used for other chronic conditions and in other jurisdictions.

Sociodemographic characteristics, prevalence of chronic conditions and sequence of condition occurrence were generally comparable across provinces, although we observed some important differences in both the prevalence of conditions and use of health services, such as services for mental health and addictions.

Several studies have reported that between 18% and 35% of people with dementia have five or more comorbid chronic conditions.^6^,^22^-^24^ We found this prevalence to be higher (38.8%–53.3%) across the four provinces, which may be driven in part by the most prevalent conditions, that is, mental illness and alcohol- or drug-induced disorders, hypertension and osteoarthritis. However, it is difficult to compare estimates across studies as there is no single definition of comorbidity and various conditions are represented, including many we did not examine (such as retinal disorders, liver disease, thyroid disease, cardiac arrhythmia, prostatic hypertrophy and insomnia).^25^-^27^

To our knowledge, few studies have examined the timing of occurrence of a broad set of chronic conditions relative to dementia. Most studies examine a narrower set of conditions and focus on vascular-related conditions as risk factors for incident dementia such as hypertension in mid-life,^28^ diabetes^29^ and stroke.^30^ Recent studies have noted that neurodegenerative conditions^31^ and hospitalization for traumatic brain injury^32^ are associated with incident dementia. We observed similar patterns of sequencing for vascular-related conditions across the provinces (most were present before dementia was ascertained). However, we found traumatic brain injury and congestive heart failure to be detected nearly as often after dementia ascertainment as before. Traumatic brain injuries increase the risk of dementia mainly when they occur in mid-life,^14^ which precedes the look-back period in our study and may explain this finding. Traumatic brain injury is also associated with falls by older people, including after the onset of dementia when mobility and coordination may be impaired.

As with other conditions measured, we could only capture data on mental illness (including depression), an established risk factor for dementia, through proxy measurement of health service use, which does not represent a clinical diagnosis. Parkinsonism, including Parkinson disease, was also more frequently ascertained prior to dementia. This is an expected pattern as Lewy body dementia can develop as Parkinson disease progresses. The increased burden of chronic conditions among people with dementia creates complex challenges in simultaneously managing health care needs for health care providers, patients and care partners.

***Strengths and limitations***

Our study presents insights from population-based data on persons living with dementia in four Canadian provinces. These data can serve as robust comparators to data from other regions.

This study has certain limitations. At a broad level, differences in the availability and structure of provincial administrative databases make comparative research across provinces and territories difficult. However, we leveraged standardized case definitions used in national disease surveillance to enhance comparisons.^1^

More specifically, while we focussed on a comprehensive set of chronic conditions with standardized definitions, we did not include other important conditions and risk factors such as smoking, alcohol consumption and physical inactivity. Second, variability in methods and database availability and completeness (e.g. differences in coding, spaces for multiple diagnoses on health claims and unobtainable emergency department data in some provinces) may have contributed to the differences observed between provinces. In addition, differences in the organization of health and social care services across provinces may explain some of the differences in prevalence of chronic conditions observed, for example, psychosocial care in QC may be more frequently provided outside of medical settings, potentially resulting in our underestimating mental illness and alcohol- or drug-induced disorders in this study. Data availability also impacted case definitions for some conditions, for example, the prevalence of traumatic brain injury may have been underreported because of a lack of available emergency department data in the participating provinces.

Third, we were limited to the case ascertainment date according to administrative databases, but administrative data case definitions do not represent formal diagnoses, and individuals may have first experienced the condition much earlier than identified. Fourth, the dementia case ascertainment algorithm, as with other health administrative data case definitions, is imperfect and may misclassify some individuals. For example, those with earlier stage dementia may be missed because of a lack of a formal diagnosis. Individuals who have not presented to the health system will also not be captured. Future studies should explore differences in comorbid chronic conditions among people with dementia across age, sex, indicators of socioeconomic status and rurality.

## Conclusion

Using national, standardized definitions for chronic conditions and a minimum common dataset approach, we compared the prevalence and sequence of occurrence of comorbid chronic conditions among people aged 65 years and older with dementia in four Canadian provinces. Our study found generally similar patterns of comorbid conditions across the provinces, suggesting that strategies for care management, resource planning and health system access could be shared across regions.

## Acknowledgements

This project was made possible through the collaboration of the Public Health Agency of Canada, ICES and the provincial governments of British Columbia, Ontario, Quebec and Prince Edward Island. Parts of this material are based on data or information compiled and provided by the Canadian Institute for Health Information (CIHI), Statistics Canada, IQVIA Solutions Canada Inc., the Ontario Ministry of Health, the Ontario Ministry of Long-Term Care and Health PEI. This study used data adapted from the Statistics Canada Postal CodeOM Conversion File, which is based on data licensed from Canada Post Corporation, and/or data adapted from the Ontario Ministry of Health Postal Code Conversion File, which contains data copied under licence from Canada Post Corporation and Statistics Canada.

The authors thank Henry Ngo and Andrea Olmstead for providing analytical support. We thank IQVIA Solutions Canada Inc. for the use of their drug information file. We thank the Toronto Community Health Profiles Partnership for providing access to the Ontario Marginalization Index.

The analyses, conclusions, opinions and statements expressed herein are solely those of the authors and do not reflect those of the funding organizations or data sources; no endorsement by the Public Health Agency of Canada, ICES, the Ontario Ministry of Health, the Ontario Ministry of Long-Term Care, Health PEI, Canadian Institute for Health Information (CIHI), Statistics Canada, IQVIA Solutions Canada Inc., the provincial governments of British Columbia, Ontario, Quebec and Prince Edward Island or the Government of Canada is intended or should be inferred.

## Funding

This study received funding from the Public Health Agency of Canada (British Columbia: 6D02303002, Ontario: 6D02303001, Quebec: 4500413802, Prince Edward Island: 4500413866). This study was also supported by ICES, which is funded by an annual grant from the Ontario Ministry of Health and the Ministry of Long-Term Care.

## Conflicts of interest

Susan E. Bronskill receives funding from the Public Health Agency of Canada (PHAC), the Canadian Institutes of Health Research (CIHR) and the Ontario Brain Institute (OBI) and support from ICES, which is funded by the Ontario Ministry of Health and the Ontario Ministry of Long-Term Care.

J. Michael Paterson receives funding from the CIHR, funding from PHAC, in support of the Canadian Chronic Disease Surveillance System (CCDSS), and support from ICES, which is funded by the Ontario Ministry of Health and the Ontario Ministry of Long-Term Care.

Andrea Gruneir receives grants from the CIHR.

Karen A.M. Phillips and Rasaq Ojasanya receive funding from PHAC via the Department of Health and Wellness, Government of Prince Edward Island.

Xibiao Ye receives funding from PHAC via the British Columbia Ministry of Health.

Kayla McLean receives funding from PHAC via the British Columbia Ministry of Health.

Victoria Massamba receives funding from PHAC via the Institut national de sant publique du Qubec (INSPQ) and the Fonds de recherche du Qubec – Sant and grants from Health Canada’s Substance Use and Addictions Program.

Isabelle Vedel receives funding from PHAC via the Institut national de sant publique du Qubec (INSPQ) and the Fonds de recherche du Qubec – Sant and grants from the CIHR.

Colleen J. Maxwell receives grants from the CIHR.

## Authors’ contributions and statement

SB: Conceptualization, methodology, validation, resources, writing – original draft, visualization, supervision, project administration, funding acquisition.

AA: Conceptualization, methodology, validation, writing – original draft, visualization.

LCM: Conceptualization, methodology, validation, writing – original draft, visualization.

XW: Methodology, software, validation, formal analysis, data curation, writing – review & editing.

HC: Methodology, validation, writing – original draft, visualization.

JMP: Conceptualization, methodology, validation, writing – review & editing.

AG: Conceptualization, methodology, validation, writing – review & editing.

KAP: Methodology, software, validation, formal analysis, data curation, writing – review & editing.

RO: Methodology, software, validation, formal analysis, data curation, writing – review & editing.

XY: Methodology, software, validation, formal analysis, data curation, writing – review & editing.

KM: Methodology, software, validation, formal analysis, data curation, writing – review & editing.

FE: Methodology, software, validation, formal analysis, data curation, writing – review & editing.

CGS: Methodology, validation, writing – review & editing.

VM: Methodology, validation, writing – review & editing.

LR: Methodology, software, validation, formal analysis, data curation, writing – review & editing.

IV: Methodology, validation, writing – review & editing.

LS: Conceptualization, methodology, validation, writing – review & editing.

CP: Conceptualization, methodology, validation, writing – review & editing.

CM: Conceptualization, methodology, validation, writing – review & editing.

The content and views expressed in this article are those of the authors and do not necessarily reflect those of the Government of Canada.

## References

[B01] Canadian Chronic Disease Surveillance System (CCDSS). Government of Canada.

[B02] Nichols E, Steinmetz JD, Vollset SE, Fukutaki K, Chalek J, Abd-Allah F (2022). Estimation of the global prevalence of dementia in 2019 and forecasted prevalence in 2050: an analysis for the Global Burden of Disease Study 2019. Nichols E, Steinmetz JD, Vollset SE, Fukutaki K, Chalek J, Abd-Allah F, et al; GBD 2019 Dementia Forecasting Collaborators.

[B03] Black W, Almeida OP (2004). A systematic review of the association between the behavioral and psychological symptoms of dementia and burden of care. Int Psychogeriatr.

[B04] Manuel DG, Garner R, s P, Bancej C, Flanagan W, Tu K, et al (2016). Alzheimer’s and other dementias in Canada, 2011 to 2031: a microsimulation Population Health Modeling (POHEM) study of projected prevalence, health burden, health services, and caregiving use. Popul Health Metr.

[B05] Melis RJ, Marengoni A, Rizzuto D, Teerenstra S, Kivipelto M, Angleman SB, Fratiglioni L (2013). The influence of multimorbidity on clinical progression of dementia in a population-based cohort. PLoS One.

[B06] Griffith LE, Gruneir A, Fisher K, Panjwani D, Gandhi S, Sheng L, et al (2016). Patterns of health service use in community living older adults with dementia and comorbid conditions: a population-based retrospective cohort study in Ontario, Canada. BMC Geriatr.

[B07] Nelis SM, Wu YT, Matthews FE, Martyr A, Quinn C, Rippon I, et al (2019). The impact of comorbidity on the quality of life of people with dementia: findings from the IDEAL study. Age Ageing.

[B08] A dementia strategy for Canada: together we aspire [Internet]. Government of Canada.

[B09] Bauer K, Schwarzkopf L, Graessel E, Holle R (2014). A claims data-based comparison of comorbidity in individuals with and without dementia. BMC Geriatr.

[B10] Tonelli M, Wiebe N, Joanette Y, Hemmelgarn BR, So H, Straus S, et al (2022). Age, multimorbidity and dementia with health care costs in older people in Alberta: a population-based retrospective cohort study. CMAJ Open.

[B11] Population estimates, quarterly: Table: 17-10-0009-01 [Internet]. Statistics Canada.

[B12] About the Canadian Chronic Disease Surveillance System (CCDSS) [Internet]. Government of Canada.

[B13] Jaakkimainen RL, Bronskill SE, Tierney MC, Herrmann N, Green D, Young J, et al (2016). Identification of physician-diagnosed Alzheimer’s disease and related dementias in population-based administrative data: a validation study using family physicians’ electronic medical records. J Alzheimers Dis.

[B14] Livingston G, Huntley J, Sommerlad A, Ames D, Ballard C, Banerjee S, et al (2020). Dementia prevention, intervention, and care: 2020 report of the Lancet Commission. Lancet.

[B15] Feely A, Lix LM, Reimer K (2017). Estimating multimorbidity prevalence with the Canadian Chronic Disease Surveillance System. Health Promot Chronic Dis Prev Can.

[B16] Dementia and stroke comorbidity among Canadians aged 65 years and older: highlights from the Canadian Chronic Disease Surveillance System [Internet]. Government of Canada.

[B17] Gargaro J, Reilly K, Perez O, Tam A, Oliveira C, Plumptre L, et al (2021). Ontario and sub regional traumatic brain injury (TBI) care report cards and provincial and regional trends in TBI Care - 2021. Ontario Neurotrauma Foundation.

[B18] Chan V, Hurst M, Petersen T, Liu J, Mollayeva T, Colantonio A, et al (2020). A population-based sex-stratified study to understand how health status preceding traumatic brain injury affects direct medical cost. PLoS One.

[B19] Boucher F, Campeau A, Champagne A, Choi B, Crain J, Draca J, et al Injury in review, 2020 edition: Spotlight on traumatic brain injuries across the life course. Public Health Agency of Canada.

[B20] Working with partners to modernize public health data [Internet]. Government of Canada.

[B21] Overview of the former expert advisory group for the pan-Canadian Health Data Strategy [Internet]. Government of Canada.

[B22] Mondor L, Maxwell CJ, Hogan DB, Bronskill SE, Gruneir A, Lane NE, et al (2017). Multimorbidity and healthcare utilization among home care clients with dementia in Ontario, Canada: a retrospective analysis of a population-based cohort. PLoS Med.

[B23] Tonelli M, Wiebe N, Straus S, Fortin M, Guthrie B, James MT (2017). Multimorbidity, dementia and health care in older people: a population-based cohort study. CMAJ.

[B24] Chen TB, Yiao SY, Sun Y, Lee HJ, Yang SC, Chiu MJ, et al (2017). Comorbidity and dementia: a nationwide survey in Taiwan. PLoS One.

[B25] Poblador-Plou B, aga A, Marta-Moreno J, Hancco-Saavedra J, Sicras-Mainar A, Soljak M, et al (2014). Comorbidity of dementia: a cross-sectional study of primary care older patients. BMC Psychiatry.

[B26] Sanderson M, Wang J, Davis DR, Lane MJ, Cornman CB, Fadden MK (2002). Co-morbidity associated with dementia. Am J Alzheimers Dis Other Demen.

[B27] Griffith LE, Gruneir A, Fisher K, Panjwani D, Gafni A, Patterson C, et al (2019). Insights on multimorbidity and associated health service use and costs from three population-based studies of older adults in Ontario with diabetes, dementia and stroke. BMC Health Serv Res.

[B28] McGrath ER, Beiser AS, DeCarli C, Plourde KL, Vasan RS, Greenberg SM, et al (2017). Blood pressure from mid- to late life and risk of incident dementia. Neurology.

[B29] Wang Y, Li C, Liang J, Gao D, Pan Y, Zhang W, et al (2023). Onset age of diabetes and incident dementia: a prospective cohort study. J Affect Disord.

[B30] Savva GM (2010). Epidemiological studies of the effect of stroke on incident dementia: a systematic review. Stroke.

[B31] Maxwell CJ, Maclagan LC, Harris DA, Wang X, Guan J, Marrie RA, et al (2022). Incidence of neurological and psychiatric comorbidity over time: a population-based cohort study in Ontario, Canada. Age Ageing.

[B32] Raj R, Kaprio J, Jousilahti P, Korja M, Siironen J (2022). Risk of dementia after hospitalization due to traumatic brain injury: a longitudinal population-based study. Neurology.

